# Supplementary material for: GlnK Facilitates the Dynamic Regulation of Bacterial Nitrogen Assimilation
Source: Biophys J. 2017 May 23;112(10):2219–30. doi: 10.1016/j.bpj.2017.04.012 (PMC5448240; doi:10.1016/j.bpj.2017.04.012)
Supplement: Document S1. Supporting Materials and Methods, Figs. S1–S7, and Tables S1 and S2 [file mmc1.pdf]

Biophysical Journal, Volume *112*

Supplemental Information

GlnK Facilitates the Dynamic Regulation of Bacterial Nitrogen Assimilation

Adam Gosztolai, Jörg Schumacher, Volker Behrends, Jacob G. Bundy, Franziska Heydenreich, Mark H. Bennett, Martin Buck, and Mauricio Barahona

Supporting Material:
GlnK facilitates the dynamic regulation of bacterial nitrogen assimilation

Adam Gosztolai[1], Jörg Schumacher[2], Volker Behrends[3,4], Jacob G Bundy[4], Franziska Heydenreich[2,†], Mark H Bennett[2], Martin Buck[2], Mauricio Barahona[1,*]

1. Department of Mathematics, Imperial College London, SW7 2AZ, London, United Kingdom.
2. Department of Life Sciences, Imperial College London, SW7 2AZ, London, United Kingdom.
3. Department of Life Sciences, University of Roehampton, SW15 5PU, London, United Kingdom.
4. Department of Surgery and Cancer, Imperial College London, SW7 2AZ, London, United Kingdom

S1 Kinetic mechanism for an enzyme with multiple alternative substrates

Here we present the detailed mathematical formulation of the kinetics of an enzyme with two alternative substrates as depicted on Fig. 4a and described by the following reactions:

$$\mathrm{GlnB} + \mathrm{E}_1 \underset{a_1}{\overset{d_1}{\rightleftharpoons}} \mathrm{E}_1\mathrm{GlnB} \xrightarrow{k_1} \mathrm{GlnB\text{-}U} + \mathrm{E}_1 \tag{S1a}$$

$$\mathrm{GlnK} + \mathrm{E}_1 \underset{a_2}{\overset{d_2}{\rightleftharpoons}} \mathrm{E}_1\mathrm{GlnK} \xrightarrow{k_2} \mathrm{GlnK\text{-}U} + \mathrm{E}_1 \tag{S1b}$$

$$\mathrm{GlnB\text{-}U} + E_2 \underset{a_3}{\overset{d_3}{\rightleftharpoons}} \mathrm{E}_2\mathrm{GlnB\text{-}U} \xrightarrow{k_3} \mathrm{GlnB} + \mathrm{E}_2 \tag{S1c}$$

$$\mathrm{GlnK\text{-}U} + \mathrm{E}_2 \underset{a_4}{\overset{d_4}{\rightleftharpoons}} \mathrm{E}_2\mathrm{GlnK\text{-}U} \xrightarrow{k_4} \mathrm{GlnK} + \mathrm{E}_2 \tag{S1d}$$

where (S1a) and (S1b) represent uridylylation, whereas (S1c) and (S1d) represent deuridylylation. Here k_1, k_2, k_3 and k_4 are the catalytic rate constants, which are functions of the glutamine concentration. Furthermore, E_1 and E_2 denote the concentration of the enzyme

[†]Current address: Laboratory of Biomolecular Research, Paul Scherrer Institute, 5232, Villigen, Switzerland and Department of Biology, ETH Zurich, 8093, Zurich, Switzerland

UT/UR with UT and UR activities respectively. To derive the rate equations corresponding to reactions (S1), we assume that complex formation between the substrates and the enzyme occurs much faster than product formation, which is the standard assumption in Michaelis-Menten kinetics (1). Furthermore, since the enzyme active sites are shared between the two substrates, we may treat either substrate as the competitive inhibitor of the other. Using techniques described in section 6.2.1 of (1) we obtain the following system of four equations:

$$v_{\mathrm{UT,GlnB}} = \frac{V_{\mathrm{UT,GlnB}}[\mathrm{GlnB}]}{K_{\mathrm{m,GlnB}}(1+[\mathrm{GlnK}]/K_{\mathrm{m,GlnK}})+[\mathrm{GlnB}]} \tag{S2a}$$

$$v_{\mathrm{UT,GlnK}} = \frac{V_{\mathrm{UT,GlnK}}[\mathrm{GlnK}]}{K_{\mathrm{m,GlnK}}(1+[\mathrm{GlnB}]/K_{\mathrm{m,GlnB}})+[\mathrm{GlnK}]} \tag{S2b}$$

$$v_{\mathrm{UR,GlnB\text{-}U}} = \frac{V_{\mathrm{UR,GlnB\text{-}U}}[\mathrm{GlnB\text{-}U}]}{K_{\mathrm{m,GlnB\text{-}U}}(1+[\mathrm{GlnK\text{-}U}]/K_{\mathrm{m,GlnK\text{-}U}})+[\mathrm{GlnB\text{-}U}]} \tag{S2c}$$

$$v_{\mathrm{UR,GlnK\text{-}U}} = \frac{V_{\mathrm{UR,GlnK\text{-}U}}[\mathrm{GlnK\text{-}U}]}{K_{\mathrm{m,GlnK\text{-}U}}(1+[\mathrm{GlnB\text{-}U}]/K_{\mathrm{m,GlnB\text{-}U}})+[\mathrm{GlnK\text{-}U}]} \tag{S2d}$$

where $K_{m,*} = (d_i + k_i)/a_i$, $i \in \{1,2,3,4\}$ are the usual Michaelis constants, and $V_{\mathrm{UT},*} = k_p[E_1]$, $p \in \{1,2\}$ and $V_{UR,*} = k_q[E_2]$, $q \in \{3,4\}$ are the maximum enzyme velocities.

In addition to the competing substrates, glutamine (GLN) may also bind to the allosteric side of UT/UR, independently of the substrate binding at a rate $K_{\mathrm{GLN}} = d_5/a_5$, where a_5 and d_5 are the association and dissociation constants of GLN. When bound, GLN activates the UR activity and inhibits the UT activity (2). We may describe this using the noncompetitive activation and inhibition model (see Section 6.2.3 and Table 6.1 of (1)), depicted in Fig. S1), which result in an additional term modifying the $V_{\mathrm{m},*}$'s of eqs. (S2):

$$v_{\mathrm{UT,GlnB}} = \frac{V_{\mathrm{UT,GlnB}}[\mathrm{GlnB}]/K_{\mathrm{m,GlnB}}}{(1+[\mathrm{GlnK}]/K_{\mathrm{m,GlnK}}+[\mathrm{GlnB}]/K_{\mathrm{m,GlnB}})}\left(\frac{1}{1+[\mathrm{GLN}]/K_{\mathrm{GLN}}}\right) \tag{S3a}$$

$$v_{\mathrm{UT,GlnK}} = \frac{V_{\mathrm{UT,GlnK}}[\mathrm{GlnK}]/K_{\mathrm{m,GlnK}}}{(1+[\mathrm{GlnB}]/K_{\mathrm{m,GlnB}}+[\mathrm{GlnK}]/K_{\mathrm{m,GlnK}})}\left(\frac{1}{1+[\mathrm{GLN}]/K_{\mathrm{GLN}}}\right) \tag{S3b}$$

$$v_{\mathrm{UR,GlnB\text{-}U}} = \frac{V_{\mathrm{UR,GlnB\text{-}U}}[\mathrm{GlnB\text{-}U}]/K_{\mathrm{m,GlnB\text{-}U}}}{(1+[\mathrm{GlnK\text{-}U}]/K_{\mathrm{m,GlnK\text{-}U}}+[\mathrm{GlnB\text{-}U}]/K_{\mathrm{m,GlnB\text{-}U}})}\left(\frac{1}{1+K_{\mathrm{GLN}}/[\mathrm{GLN}]}\right) \tag{S3c}$$

$$v_{\mathrm{UR,GlnK\text{-}U}} = \frac{V_{\mathrm{UR,GlnK\text{-}U}}[\mathrm{GlnK\text{-}U}]/K_{\mathrm{m,GlnK\text{-}U}}}{(1+[\mathrm{GlnB\text{-}U}]/K_{\mathrm{m,GlnB\text{-}U}}+[\mathrm{GlnK\text{-}U}]/K_{\mathrm{m,GlnK\text{-}U}})}\left(\frac{1}{1+K_{\mathrm{GLN}}/[\mathrm{GLN}]}\right). \tag{S3d}$$

S2 Kinetic mechanism for an enzyme with competing allosteric effectors

The mathematical description of simple inhibition and activation can be found in standard textbooks on reaction (1). However, enzymes with multiple competing allosteric effectors such as AT/AR are rare, therefore we need to give a derivation of such a system. Fig. 4b illustrates graphically an enzyme which can bind two allosteric effectors (inhibitors or

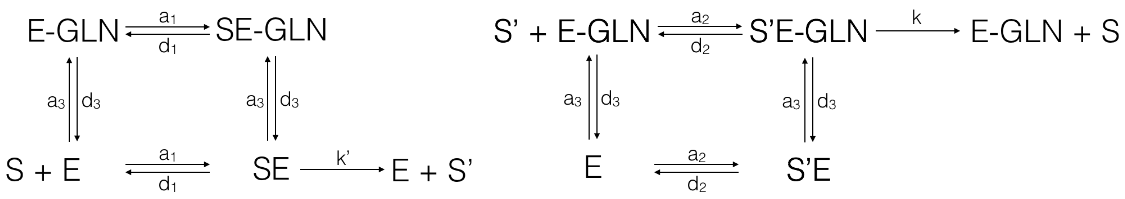

Figure S1: Mechanism for allosteric regulation of UT/UR by glutamine (GLN) based on Fig. 4a. On the left hand side glutamine inhibits the conversion of the substrate S (GlnB, GlnK) to S' (GlnB-U, GlnK-U), whereas on the right hand side GLN activates the conversion of S' to S. It is assumed that the substrate binding is unaffected by the presence of GLN.

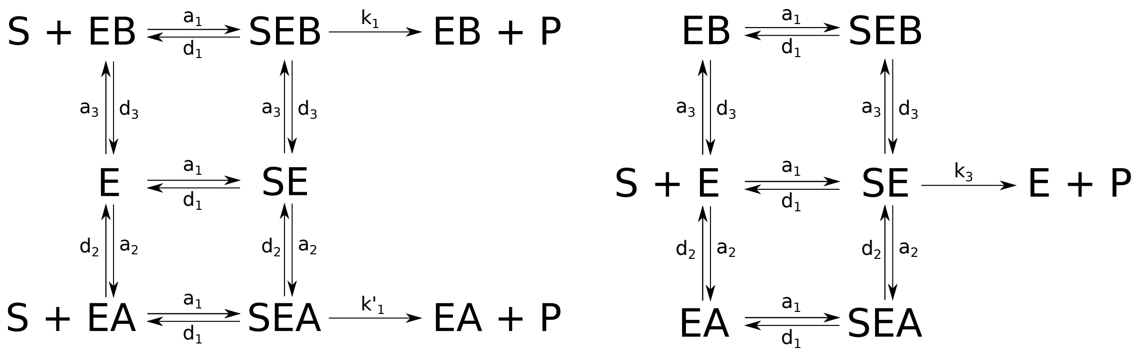

Figure S2: Mechanism for allosteric regulation of AT/AR by competitive effectors based on Fig. 4b. On the left hand side proteins A and B compete for activation of enzyme E, whereas on the right hand side they compete for inhibition of enzyme E. It is assumed that the substrate binding is unaffected by the presence of the effector.

activators) and a substrate molecule simultaneously, but their presence do not influence each others' binding. As in Michaelis-Menten kinetics, we assume that the reactions involving complex formation between the enzyme, the substrates and the effectors are in equilibrium independently and at a much shorter timescale than that at which the reaction takes place.

We begin with the case of multiple competing allosteric activators, which can be depicted by the reaction diagram on the left of Fig. S2. Let E denote a general enzyme catalysing the conversion of a substrate S to a product P. Assume that E is in a neutral state unless one of the activators A or B (having different affinities) bind to the common allosteric site. When either effector is bound, product formation may take place, however the reaction rate depends on whether A or B is bound. To express the reaction rate mathematically as a function of effector concentrations note that we can define five different complexes whose concentrations are denoted as: $c_1 = [SE]$, $c_2 = [SEA]$, $c_3 = [EA]$, $c_4 = [EB]$, $c_5 = [SEB]$.

Also, let $s = [S]$, $a = [A]$, $b = [B]$. Then by the law of mass action it follows that:

$$(e_0 - c_1 - c_2 - c_3 - c_4 - c_5)s = K_1 c_1 \tag{S4a}$$
$$(e_0 - c_1 - c_2 - c_3 - c_4 - c_5)a = K_2 c_3 \tag{S4b}$$
$$(e_0 - c_1 - c_2 - c_3 - c_4 - c_5)b = K_3 c_4 \tag{S4c}$$
$$c_3 s = K_1 c_2 \tag{S4d}$$
$$c_4 s = K_1 c_5 \tag{S4e}$$
$$c_1 a = K_2 c_2 \tag{S4f}$$
$$c_1 b = K_3 c_5 \tag{S4g}$$

where $e_0 = e + c_1 + c_2 + c_3 + c_4 + c_5$ is the total concentration of the enzyme and $K_i = d_i/a_i$. This is a linear system of equations with eight variables. There are seven equations, but two are linear combinations of the other five (system has rank 5) so we can express the concentration of the complexes as functions of three, which we choose to be s, a and b. Substituting (S4d), (S4e), (S4f) and (S4g) into (S4a) we obtain:

$$\left(e_0 - \frac{K_2}{a}c_2 - c_2 - \frac{K_1}{s}c_2 - \frac{K_1}{s}\frac{b}{K_3}\frac{K_2}{a}c_2 - \frac{b}{K_3}\frac{K_2}{a}c_2\right) s = K_2\frac{K_1}{a}c_2$$
$$\left(e_0 - \frac{K_3}{b}c_5 - \frac{a}{K_2}\frac{K_3}{b}c_5 - \frac{K_1}{s}\frac{a}{K_2}\frac{K_3}{b}c_5 - \frac{K_1}{s}s_5 - c_5\right) a = K_3\frac{K_1}{b}c_5.$$

Rearranging, it follows that:

$$c_2 = \frac{e_0}{\left(1 + \frac{K_1}{s}\right)\left(1 + \frac{K_2}{a} + \frac{b}{K_3}\frac{K_2}{a}\right)}$$
$$c_5 = \frac{e_0}{\left(1 + \frac{K_1}{s}\right)\left(1 + \frac{K_3}{b} + \frac{a}{K_2}\frac{K_3}{b}\right)}.$$

Hence the catalytic rate of the enzyme is:

$$v = k_1 c_2 + k_1' c_5 = \frac{1}{\left(1 + \frac{K_1}{s}\right)}\left[\frac{k_1 e_0}{\left(1 + \frac{K_2}{a} + \frac{b}{K_3}\frac{K_2}{a}\right)} + \frac{k_1' e_0}{\left(1 + \frac{K_3}{b} + \frac{a}{K_2}\frac{K_3}{b}\right)}\right]. \tag{S5}$$

Hence comparing with the case of a single activator (see Cornish-Bowden 2013, pg. 152-157) we obtain that there the effect of the two activators are almost additive except for an additional mixed term in the denominator.

We proceed similarly to obtain a relationship for the case of multiple competing allosteric inhibitors (see right of Fig. S2). Using rapid equilibrium assumption we obtain a set of equations identical to system (S4). Here we need to solve for complex c_1, so we write:

$$\left(e_0 - c_1 - \frac{b}{K_3}c_1 - \frac{K_1}{s}\frac{b}{K_3}c_1 - \frac{K_1}{s}\frac{a}{K_2}c_1 - \frac{a}{K_2}c_1\right) s = K_1 c_1,$$

from which it follows that the enzyme velocity is:

$$v = k_1 c_1 = \frac{e_0}{\left(\frac{K_1}{s} + 1\right)\left(1 + \frac{a}{K_2} + \frac{b}{K_3}\right)}. \tag{S6}$$

So similarly to the case of single inhibitor we obtain that there is a reduction of the maximum velocity of the reaction leaving the K_1 unchanged.

S3 Global sensitivity analysis of the model

To assess the significance of the model parameters we conducted a sensitivity analysis. The objective of the latter is to quantify the change in the system output to a small perturbation in one or a combination of parameters, while the others are kept constant. Since the outputs are time series, the distance between two time series if of interest, therefore conventional (local) sensitivity analysis techniques, which are only concerned with variations in the steady state are not adequate. Instead, here we use the eFAST method (3) to assess parameter sensitivities, which is a global sensitivity analysis technique, and is available in the Systems Biology Toolbox 2 for Matlab (4). In essence, global sensitivity analyses are concerned with sampling the parameter space - usually randomly, according to some optimal strategy - in the vicinity of the fitted parameters and estimating the resulting variation in the output. The latter is usually measured by the pointwise difference between the nominal and perturbed output time series integrated over the time range of interest. The eFAST method in particular samples the parameter space along such a trajectory that allows the model output to be expressed as a Fourier series. Then the variance in the output can be decomposed into a sum of terms involving Fourier coefficients, which can be estimated using Monte Carlo techniques. In our analysis we used a 10% perturbation from the nominal parameters and 10^5 samples.

Fig. S3 shows the relative sensitivities of the model parameters. Since GlnB/GlnB-U and GlnK/GlnK-U are important effectors of the AT/AR enzyme, GS-A levels will depend on the parameters that are specific to uridylylation. However, GS-A levels do not feed back into the model since we used glutamine concentration as a driver. Hence the parameters specific to adenylylation do not affect GlnB-U and GlnK-U states. Hence we grouped the parameters according to whether they are involved in uridylylation or adenylylation. As expected, the most sensitive parameters are the maximum enzyme velocities, Michaelis constants and the Hill-coefficient of the AT/AR reactions (n_{AT}), which has a large effect on setting the basal GS-A levels. Another parameter of high sensitivity is the percentage sequestration, x, which confirms the importance of modelling AmtB-GlnK complex formation. The parameters of lowest relative sensitivity are the activation constants K_{GlnB}, $K_{\mathrm{GlnB\text{-}U}}$, hence we fixed these from the literature (see Table I in the paper). Although $K_{\mathrm{GlnK\text{-}U}}$ was also found to be relatively insensitive, which is commensurate with the wide sample distribution in Fig. S4, we could not find parameters in the literature measured under similar conditions to our experiments. We leave these at their fitted values, because they are highly influential for our predictions.

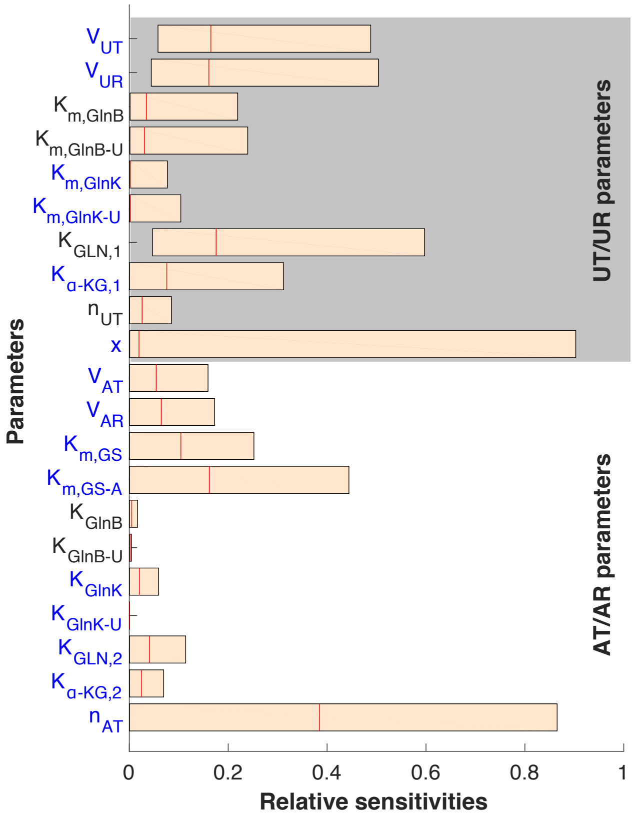

Figure S3: Relative sensitivity of variables to model parameters obtained using eFAST global sensitivity analysis (3, 4). The fitted parameters are marked in blue. Bars show the maximum and minimum sensitivity of the individual variables (GlnB-U, GlnK-U and GS-A) obtained for 10^5 samples from the parameter space with a maximum 10% deviation from the fitted parameters. Red line indicates the median value.

S4 Evolutionary Monte Carlo optimisation algorithm for parameter fitting

The parameters were fitted using the 'Squeeze and Breathe' evolutionary optimisation method (5). Let $\mathbf{X}(t) = \{x_1(t), \ldots, x_d(t)\}$ denote the state of the system with d variables at time t, which in our case are the concentrations of GlnB, GlnK and GS. The evolution of these variables is described by a set of ODEs, $\dot{\mathbf{X}} = f(\mathbf{X}, t; \theta)$, where $\theta = \{\theta_1, \ldots \theta_n\}$ is a set of n parameters. In essence, the objective of the parameter optimisation is to find parameters θ such that the distance between the solution $\mathbf{X}$ and an experimental dataset $\mathcal{D} = \{\widetilde{\mathbf{X}}(t_i) | i = 1, \ldots, m\}$ of m observations is minimised. To account for measurement error we define the following cost function, weighted by the standard error of the measurements:

$$E_{\mathcal{D}}(\theta) = \min_{\theta} \left\{ \sum_{j=1}^{m} \|(\mathbf{X}(t_i; \theta) - \widetilde{\mathbf{X}}(t_i))/SE_i\| \right\},$$

where $\|.\|$ is the Euclidean norm. Since the cost function depends on all n parameters, its value lies in an n dimensional space. The optimal parameters will be the coordinates corresponding the global minimum of $E_{\mathcal{D}}(\theta)$ over all possible parameters. The cost function $E_{\mathcal{D}}(\theta)$ defines a very rough landscape and as a result optimisation methods may get stuck in a local minimum. In fact, an algorithm which guarantees to find the best parameters does not exist. Therefore the objective of the parameter optimisation is to explore a large portion of this space to get as close to the global minimum as possible. The Squeeze and Breathe algorithm achieves this by first running local optimisation around random samples in the parameter space. These are then ranked according to optimality and culled keeping only the best few. The culled set is then used to obtain a posterior sampling distribution. The process is repeated until the difference between subsequent posterior distributions is small.

The fitted parameters are shown in Table I in the main paper and the histograms from the parameter sampling are shown in Fig. S4. The histograms show the number of times the parameter fitting algorithm converged to the particular parameter values, whereas the red asterisk show the parameter value with the minimum cost function. A narrow distribution around a fitted parameter reflects a well defined minimum in the explored parameter range, indicating that the fitted model has higher sensitivity to the variations of these parameters. On the other hand a wide distribution shows that the cost function landscape is shallow or contains many local minima. This indicates that the model might be less sensitive to the corresponding parameters. Fig. S4 show that most parameter have a narrow spread. An exception is $K_{\text{GlnK-U}}$, which show high variation, indicating low sensitivity of the model to this parameter (confirmed by sensitivity analysis). In the main paper we argue, that the low sensitivity of $K_{\text{GlnK-U}}$ in the WT model has biological origins, since the GlnK protein is less potent compared to GlnB. However, we do not $K_{\text{GlnK-U}}$ to a value found in the literature, since this parameter will be essential for accurate prediction of the GS adenylylation levels in the mutant strain containing no GlnB. The parameters to be fitted were chosen also based on the information provided by a global sensitivity analysis of the model presented in Section S3.

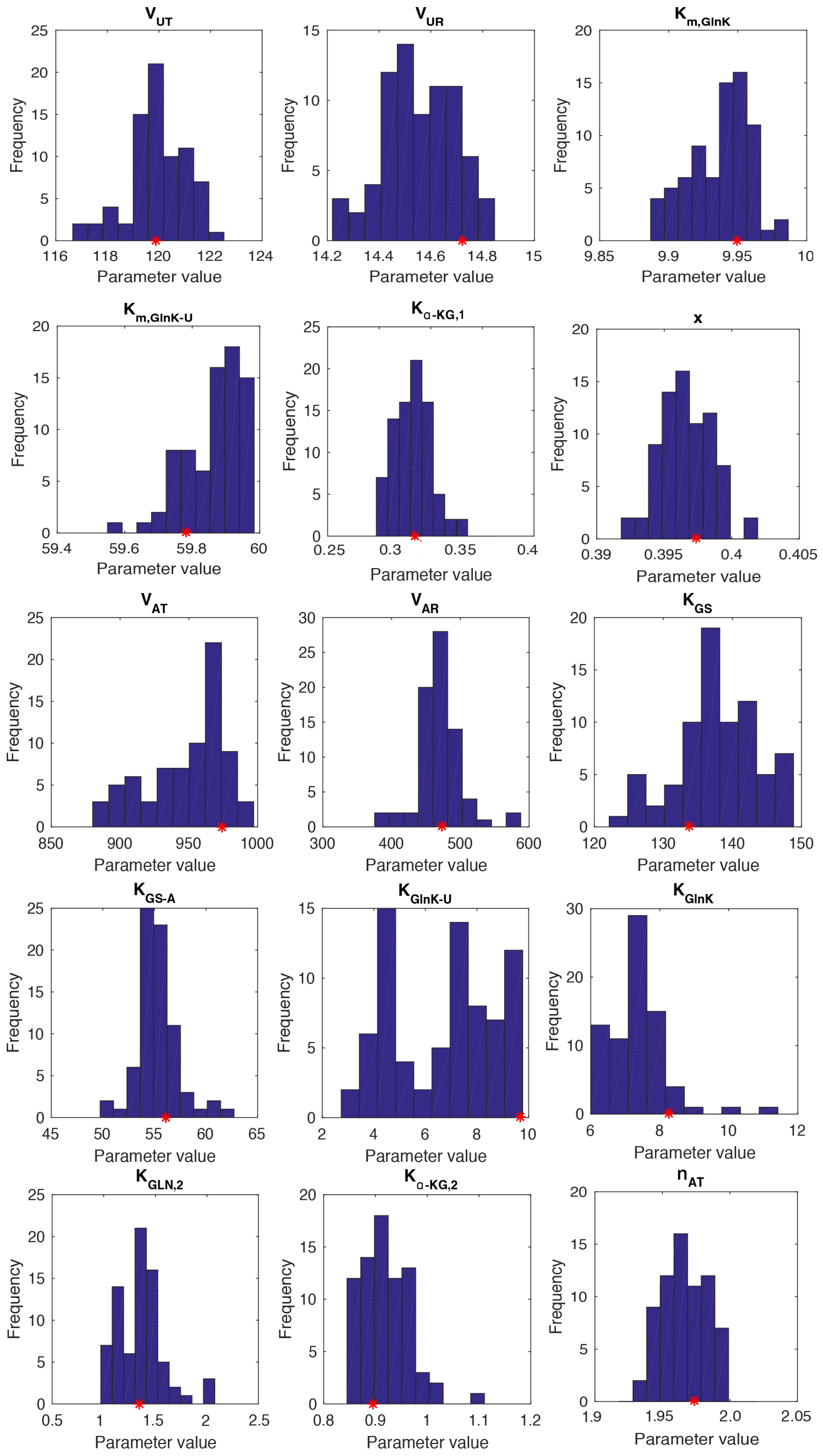

Figure S4: Histograms of fitted parameters obtained through the Squeeze-and-Breathe algorithm (5). Red asterisks indicate the parameter combination with the lowest cost function.

S5 Model selection: uridylylation reactions with and without sequestration

We compared the uridylylation models with and without GlnK sequestration (Eq. 4 in the main text) using different model selection criteria. All criteria unanimously selected the model with sequestration.

Our model is fitted to the WT time-series (training dataset). The fitted model is then used to predict the dynamical responses for the two mutants (test datasets) without using the measurements. The model with sequestration provides an improved fit both for the training dataset and an improved prediction of the test datasets, yet it contains 6 parameters (instead of 4 parameters for the model without sequestration).

To make the comparison precise, we obtain the statistical significance of the models with and without sequestration based on two information criteria:

- Akaike Information Criterion corrected for small datasets:

$$\mathrm{AICc} = n \log(\mathrm{RSS}) + \frac{2kn}{n-k-1}$$

- Bayesian Information Criterion:

$$\mathrm{BIC} = n \log(\mathrm{RSS}) + k \log(n)$$

Here n is the number of data points (24 using the WT and the $\Delta glnB$ datasets); k is the number of fitted parameters (4 without sequestration and 6 with sequestration); and RSS is the residual sum-of-squares deviation of the model from the data (Eq. 7).

Both criteria compare models based on their goodness-of-fit and penalise the number of parameters. The AICc criterion is theoretically more appropriate (since it compares the model to the true model), but has a bias toward models with higher complexity. As a further confirmation, we also computed the BIC criterion, which puts a higher penalty on the number of model parameters, and would thus favour the lower complexity model.

Table S1 shows that the model with sequestration is selected according to both the AICc and BIC criteria, as shown by lower values of both criteria for the model with sequestration.

Table S1: Information criteria for model selection of uridylylation model with and without sequestration using the WT and $\Delta glnB$ data ($n = 24$ points)

**Model**	**Number of fitted parameters (k)**	**RSS**	**AICc**	**BIC**
without sequestration	4	60.3	108.5	111.1
with sequestration	6	21.7	**90.8**	**92.9**

S6 Comparison to the Straube model of GlnB uridylylation

The enzyme UT/UR has two active sites (6), catalysing uridylylation (UT) of GlnB/GlnK and deuridylylation (UR) of GlnB-U and GlnK-U. Having two active sites both with two distinct substrates means there are four different ways in which ternary complexes can form. To test whether these contribute significantly to product formation in the UT and UR reactions we compared the Straube model (7), which describes GlnB uridylylation and includes the ternary complex between UT/UR, GlnB and GlnB-U, and another model, where the contribution of the ternary complex is ignored. The latter is equivalent to the classical Goldbeter-Koshland model (8) that assumes Michaelis-Menten kinetics with allosteric inhibition and activation by glutamine for the UR and UT reactions respectively. For both models we used literature parameters from the *in vitro* reconstituted GlnB-UT/UR system (9) except for the V_{max}, which had to be fitted. To make the best comparison we used the fact that $V_{\mathrm{max}} = ke$, where k is the catalytic rate and e is the concentration of the enzyme, fixed the ratio between $V_{UT}/V_{UR} = k_{UT}/k_{UR}$ using literature values of k_{UT} and k_{UR} (9) and fitted only one parameter, corresponding to e. As Fig. S5 shows, both models produce indistinguishable results under most conditions except 30s after upshift where the Straube model, being a steady state model, performs worse due to strong transient dynamics. This suggests that the contribution of the ternary complexes are not likely to be significant.

Despite performing well on GlnB uridylylation using literature parameters, the Straube model could only produce an adequate fit to the GlnK data in the $\Delta glnB$ strain when the basal UT activity (which is one of its parameters) was as high as 50%. We found this after an extensive parameter search using a non-linear least squares fitting procedure. This is much higher than the 1% basal UT activity reported in a previous in vitro study with GlnB and UT/UR (9). Furthermore, due to the number of complexes this model cannot be extended to treat GlnB and GlnK at the same time.

The high basal UT activity for GlnK in the Straube model highlights the asymmetry in the system with respect to glutamine. In other words, glutamine binding to UT/UR activates the UR activity and inhibits the UT activity by different relative amounts. We could account for this in our model by defining two different K_d for the activation of UR and inhibition of UT reactions by glutamine.

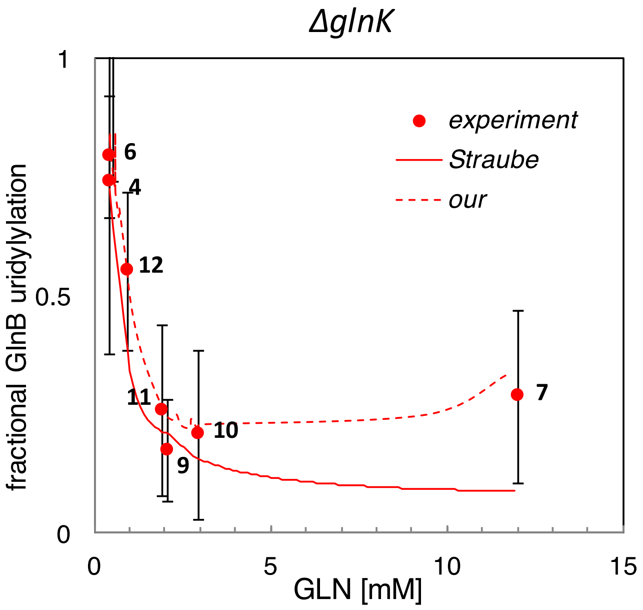

Figure S5: Measured and fitted dynamics of fractional GlnB uridylylation against glutamine for our model and the Straube model, normalised to total concentrations (Fig. 3 in paper). Error bars represent mean squared error of two independent measurements (total and modified protein) with three replicates each. For both figures and models we used parameters from the in vitro reconstituted GlnB-UT/UR system. Numbers correspond to sample points on other figures. Both models can fit the data equally well for all glutamine conditions in the $\Delta glnK$ strain except 30s after ammonium upshift (point 7) where the steady state Straube model underestimates GlnB uridylylation.

S7 Additional Supplementary Figures and Tables

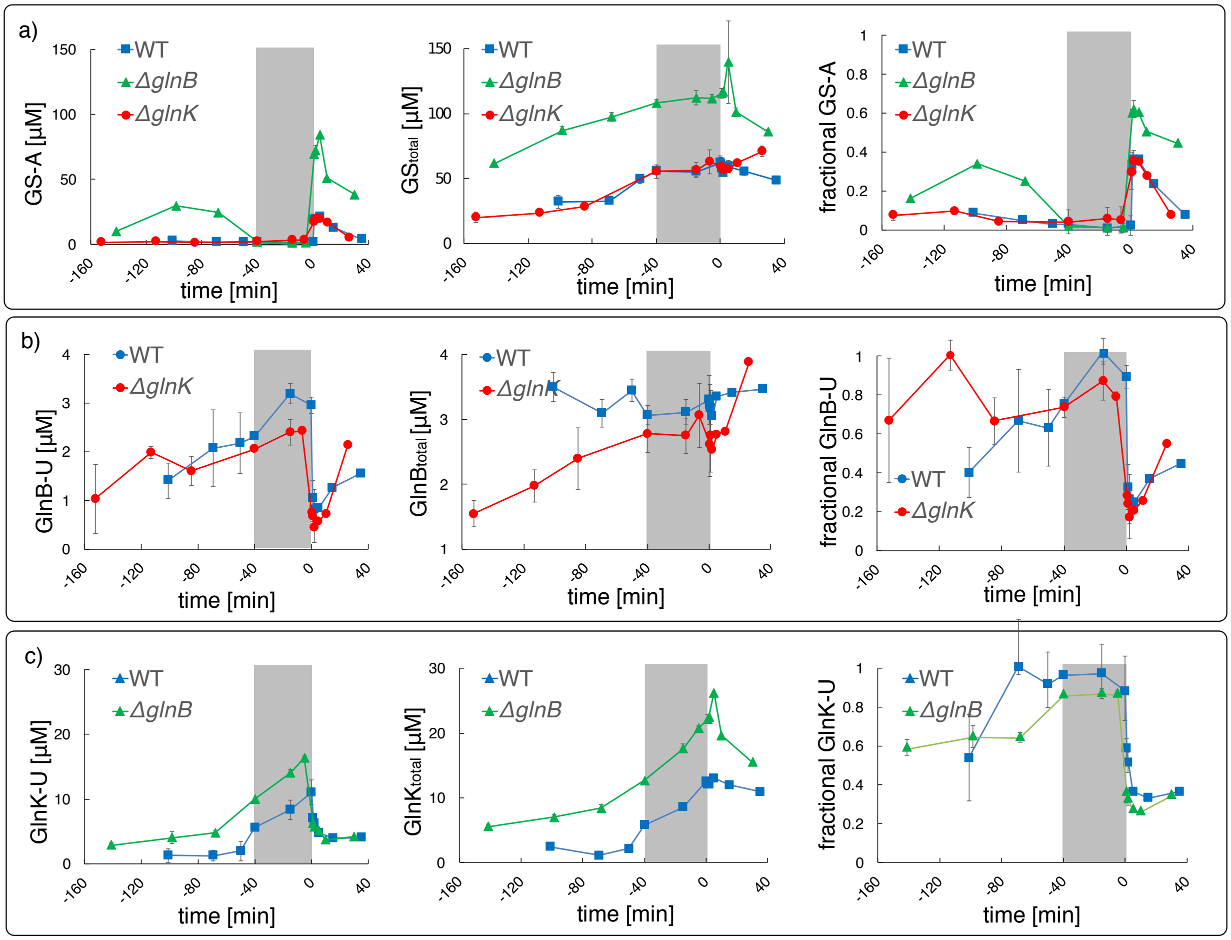

Figure S6: PTM state, total concentration and fractional PTM state of a) GS b) GlnB and c) GlnK proteins during run-out, starvation and a subsequent ammonium upshift at $t = 0s$. Starvation is marked by the grey area. The symbols and error bars represent averages ±s.e (n=3). The lines are guides to the eye.

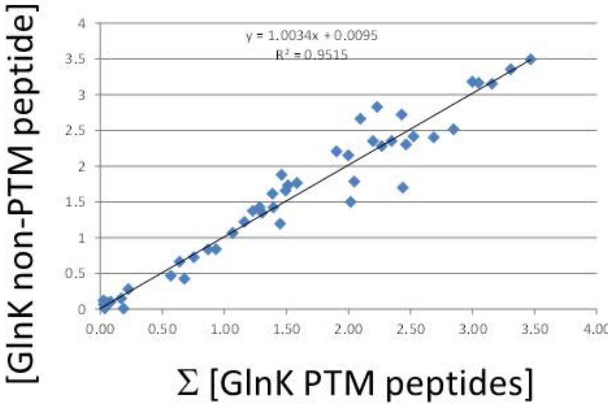

Figure S7: Correlation between GlnK concentration derived from non-uridylylated peptides and that derived independently from the sum of uridylylated and non-uridylylated peptide GAEYSVNFLPK.

Table S2: MRM-MS signals of GlnK unlabelled/labelled signature peptides

**Protein**	**Peptide**	**Internal standard (Y/N)**	**Q1**	**Q3**	**Retention**	**Collision energy**
GlnK-1	GAEYSVNFLPK-1a	N	612.8	804.5	35.8	30
GlnK-1	GAEYSVNFLPK-1b	N	612.8	967.5	35.8	30
GlnK-1	GAEYSVNFLPK-1c	N	612.8	244.2	35.8	30
GlnK-1	GAEYSVNFLPK-1a-is	Y	616.8	812.5	35.8	30
GlnK-1	GAEYSVNFLPK-1b-is	Y	616.8	975.5	35.8	30
GlnK-1	GAEYSVNFLPK-1c-is	Y	616.8	252.2	35.8	30
GlnK(u)-1	GAEY(U)SVNFLPK-1a	N	765.9	244.2	33.2	30
GlnK(u)-1	GAEY(U)SVNFLPK-1b	N	765.9	804.5	33.2	30
GlnK(u)-1	GAEY(U)SVNFLPK-1a-is	Y	769.9	252.2	33.2	30
GlnK(u)-1	GAEY(U)SVNFLPK-1b-is	Y	769.9	812.5	33.2	30
GlnK-2	IFVAELQR-2a	N	488.3	715.4	31.8	30
GlnK-2	IFVAELQR-2b	N	488.3	616.3	31.8	30
GlnK-2	IFVAELQR-2c	N	488.3	862.5	31.8	30
GlnK-2	IFVAELQR-2a-is	Y	493.3	725.4	31.8	30
GlnK-2	IFVAELQR-2b-is	Y	493.3	626.3	31.8	30
GlnK-2	IFVAELQR-2c-is	Y	493.3	872.5	31.8	30

Supporting References

[1] Cornish-Bowden, A., 2013. Fundamentals of enzyme kinetics. John Wiley & Sons, Weinheim, Germany.

[2] Jiang, P., J. A. Peliska, and A. J. Ninfa, 1998. Enzymological characterization of the signal-transducing uridylyltransferase/uridylyl-removing enzyme (EC 2.7. 7.59) of *Escherichia coli* and its interaction with the PII protein. *Biochem.* 37:12782–12794.

[3] Saltelli, A., S. Tarantola, and K.-S. Chan, 1999. A quantitative model-independent method for global sensitivity analysis of model output. *Technometrics* 41:39–56.

[4] Schmidt, H., and M. Jirstrand, 2006. Systems Biology Toolbox for MATLAB: a computational platform for research in systems biology. *Bioinformatics* 22:514–515.

[5] Beguerisse-Díaz, M., B. Wang, R. Desikan, and M. Barahona, 2012. Squeeze-and-breathe evolutionary Monte Carlo optimization with local search acceleration and its application to parameter fitting. *J. R. Soc. Interface* 9:1925–1933.

[6] Zhang, Y., E. L. Pohlmann, J. Serate, M. C. Conrad, and G. P. Roberts, 2010. Mutagenesis and Functional Characterization of the Four Domains of GlnD, a Bifunctional Nitrogen Sensor Protein. *J. Bacteriol.* 192:2711–2721.

[7] Straube, R., 2013. Sensitivity and robustness in covalent modification cycles with a bifunctional converter enzyme. *Biophys. J.* 105:1925–1933.

[8] Goldbeter, A., and D. E. Koshland, 1981. An amplified sensitivity arising from covalent modification in biological systems. *Proc. Natl. Acad. Sci. USA* 78:6840–6844.

[9] Ventura, A. C., P. Jiang, L. Van Wassenhove, D. Del Vecchio, S. D. Merajver, and A. J. Ninfa, 2010. Signaling properties of a covalent modification cycle are altered by a downstream target. *Proc. Natl. Acad. Sci. USA* 107:10032–10037.
